# Supplementary material for: Conflicting Nongenomic Effects of Progesterone in the Myometrium of Pregnant Rats
Source: Int J Mol Sci. 2021 Feb 22;22(4):2154. doi: 10.3390/ijms22042154 (PMC7926872; doi:10.3390/ijms22042154)
Supplement: Supplementary file 1 [file ijms-22-02154-s001.pdf]

Supplementary Information for
Conflicting non-genomic effects of progesterone in the myometrium of pregnant rats

Katsuhiko Yasuda*, Aya Yoshida, Hidetaka Okada

Department of Obstetrics and Gynecology, Kansai Medical University, 2-5-1 Shinmachi, Hirakata, Osaka 573-1010, Japan

*Katsuhiko Yasuda
Email: ksafety@yasuda-mf.or.lp

This PDF file includes:

Figures S1
Tables S1 to S6

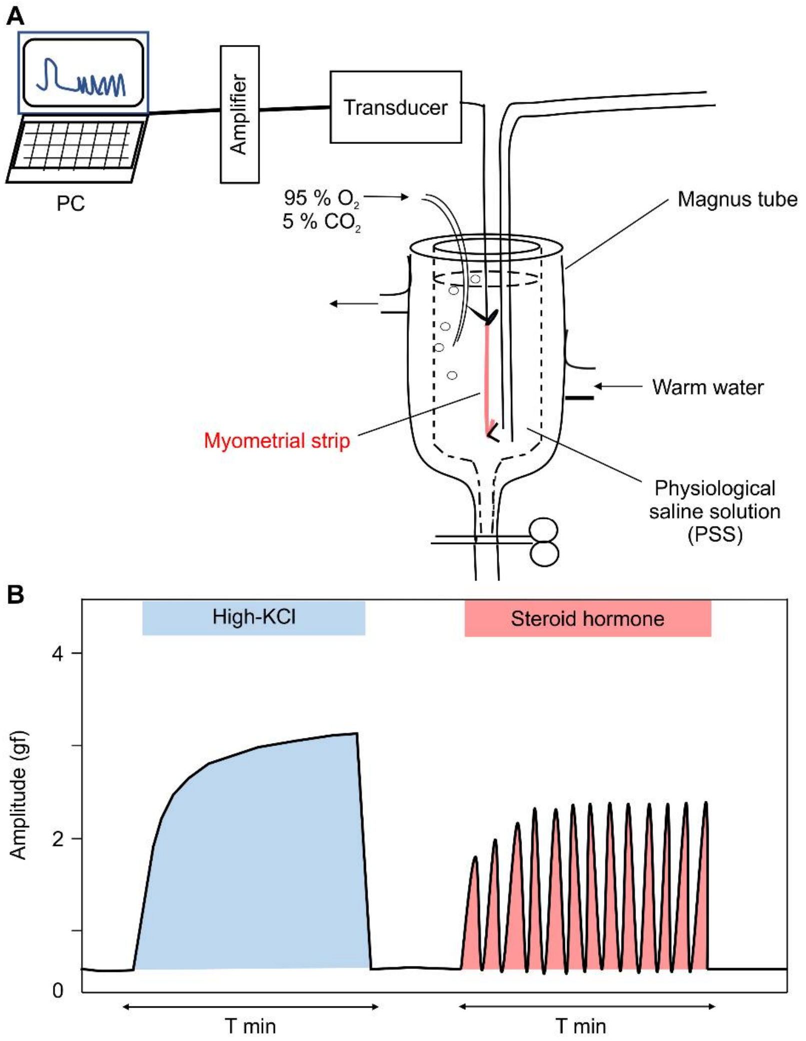

Figure S1. Experimental measuring system and evaluation of hormone-induced contraction. (A) A myometrial strip attached to a holder under 1 g resting tension in a Magnus tube filled with physiological saline solution (PSS). The contractile activity was recorded using a force-displacement transducer connected to a strain amplifier and analyzed using the Unique Acquisition software package. (B) Representative pattern of high-KCL-induced contraction and hormone-induced contraction. High-KCl-induced peak area for 5 or 10 min was defined as the reference area. Contractile activity, including tension, frequency, and contractile time, was evaluated as the relative ratio of peak area (RRPA: hormone-induced peak area/high-KCl-induced peak area) because the absolute values of peak areas varied between the individual strips even when prepared from the same uterine tissue.

Table S1

Relative ratio of the peak areas of natural steroid hormone-induced contraction

Concentration (M)<br><br>Group (n = 6, mean ± SD)	10^{-7}	5 x 10^{-7}	10^{-6}	5 x 10^{-6}	10^{-5}	5 x 10^{-5}	10^{-4}
P_4	0.01 ± 0.03	0.18 ± 0.14	0.29 ± 0.17	0.46 ± 0.12	0.41 ± 0.08	0.07 ± 0.04	-0.00 ± 0.01
E_2	-0.01 ± 0.01	0.00 ± 0.02	0.01 ± 0.03	0.02 ± 0.03	0.00 ± 0.01	0.00 ± 0.01	0.00 ± 0.02
T	0.00 ± 0.01	0.00 ± 0.02	0.00 ± 0.01	-0.01 ± 0.01	0.03 ± 0.07	0.03 ± 0.08	0.00 ± 0.01
Cortisol	0.00 ± 0.00	0.00 ± 0.01	0.01 ± 0.02	0.01 ± 0.02	0.00 ± 0.01	0.00 ± 0.00	0.02 ± 0.04
Aldosterone	0.00 ± 0.01	0.00 ± 0.01	0.01 ± 0.01	0.00 ± 0.00	0.01 ± 0.02	0.00 ± 0.01	0.02 ± 0.04
Vehicle	-0.00 ± 0.02	-0.00 ± 0.01	-0.00 ± 0.01	0.00 ± 0.01	0.00 ± 0.01	-0.01 ± 0.01	-0.01 ± 0.01

Table S2

Relative ratio of the peak areas of progesterone-induced contraction at various concentrations

Time (min)<br>Group (n = 6, mean ± SD)	-5-0	0-5	5-10	10-15	15-20
Vehicle	0.00 ± 0.00	0.00 ± 0.01	-0.00 ± 0.01	-0.01 ± 0.01	0.00 ± 0.01
P_4: 10^{-7} M	0.00 ± 0.00	-0.00 ± 0.01	-0.00 ± 0.01	0.00 ± 0.00	-0.00 ± 0.01
P_4: 5 x10^{-7} M	0.00 ± 0.00	0.08 ± 0.11	0.13 ± 0.18	0.15 ± 0.17	0.22 ± 0.13
P_4: 10^{-6} M	0.00 ± 0.00	0.07 ± 0.09	0.18 ± 0.15	0.28 ± 0.09	0.31 ± 0.04
P_4: 5 x10^{-6} M	0.00 ± 0.00	0.21 ± 0.05	0.31 ± 0.06	0.32 ± 0.06	0.33 ± 0.06
P_4: 10^{-5} M	0.00 ± 0.00	0.24 ± 0.16	0.34 ± 0.18	0.30 ± 0.16	0.19 ± 0.13
P_4: 5 x10^{-5} M	0.00 ± 0.00	0.29 ± 0.15	0.05 ± 0.06	0.03 ± 0.04	0.02 ± 0.04
P_4: 10^{-4} M	0.00 ± 0.00	0.30 ± 0.13	0.01 ± 0.07	0.01 ± 0.01	0.00 ± 0.03

Table S3

Relative ratio of the peak areas of oxytocin-induced contraction and high-KCl-induced contraction in the progesterone and vehicle groups

P_4 (M)<br>Group (n = 6, mean ± SD)	0	10^{-7}	5 x 10^{-7}	10^{-6}	5 x 10^{-6}	10^{-5}	5 x 10^{-5}	10^{-4}
Oxytocin + P_4	0.26 ± 0.14	0.36 ± 0.12	0.38 ± 0.10	0.41 ± 0.10	0.47 ± 0.10	0.45 ± 0.16	0.08 ± 0.09	0.00 ± 0.01
Oxytocin + Vehicle	0.23 ± 0.12	0.30 ± 0.12	0.27 ± 0.12	0.25 ± 0.10	0.23 ± 0.10	0.23 ± 0.08	0.23 ± 0.08	0.24 ± 0.07
High-KCl + P_4	1.02 ± 0.03	1.09 ± 0.14	1.04 ± 0.16	0.96 ± 0.16	0.73 ± 0.16	0.37 ± 0.13	-0.01 ± 0.05	-0.09 ± 0.07
High-KCl + Vehicle	1.03 ± 0.03	1.24 ± 0.10	1.28 ± 0.11	1.28 ± 0.11	1.25 ± 0.11	1.21 ± 0.12	1.16 ± 0.13	1.10 ± 0.15

Table S4

Relative ratio of the peak areas of progesterone-induced contraction and high-KCl-induced contraction in the RU486 and vehicle groups

Time (min)<br><br>Group (n = 6, mean ± SD)	-5-0	0-5	5-10	10-15	15-20
RU486-pretreatment + P_4 (5 x 10^{-6} M)	0.00 ± 0.00	0.29 ± 0.19	0.41 ± 0.16	0.35 ± 0.14	0.32 ± 0.14
Vehicle-pretreatment + P_4 (5 x 10^{-6} M)	0.00 ± 0.00	0.34 ± 0.17	0.46 ± 0.10	0.44 ± 0.07	0.42 ± 0.09
RU486-pretreatment + P_4 (5 x 10^{-5} M)	0.00 ± 0.00	0.21 ± 0.06	0.01 ± 0.03	0.01 ± 0.01	0.00 ± 0.00
Vehicle-pretreatment + P_4 (5 x 10^{-5} M)	0.00 ± 0.00	0.23 ± 0.09	0.05 ± 0.04	0.00 ± 0.02	-0.00 ± 0.01
High-KCl + RU486-pretreatment + P_4 (5 x 10^{-6} M)	1.08 ± 0.16	0.86 ± 0.17	0.66 ± 0.17	0.53 ± 0.15	0.46 ± 0.14
High-KCl + Vehicle-pretreatment + P_4 (5 x 10^{-6} M)	1.07 ± 0.03	0.90 ± 0.04	0.70 ± 0.04	0.57 ± 0.04	0.49 ± 0.05

Table S5

Relative ratio of the peak areas of progesterone-induced contraction under treatment with physiological saline solution (PSS) or calcium-free PSS and in vehicle-pretreatment, verapamil-pretreatment, and nifedipine-pretreatment groups

Time (min)<br><br>Group (*n* = 6, mean ± SD)	-5-0	0-5	5-10	10-15	15-20
P_4 (5 x 10^{-6} M) in PSS	0.00 ± 0.00	0.09 ± 0.09	0.25 ± 0.10	0.28 ± 0.04	0.27 ± 0.02
P_4 (5 x 10^{-6} M) in calcium-free PSS	0.00 ± 0.00	-0.01 ± -0.01	-0.01 ± -0.01	-0.01 ± -0.01	-0.01 ± -0.01
Vehicle-pretreatment + P_4 (5 x 10^{-6} M)	0.00 ± 0.00	0.23 ± 0.16	0.28 ± 0.16	0.32 ± 0.11	0.33 ± 0.10
Verapamil (5 x 10^{-7} M)-pretreatment + P_4 (5 x 10^{-6} M)	0.00 ± 0.00	0.00 ± -0.01	-0.01 ± 0.01	-0.00 ± 0.01	0.00 ± 0.02
Nifedipine (10^{-7} M)-pretreatment + P_4 (5 x 10^{-6} M)	0.00 ± 0.00	0.01 ± 0.03	-0.01 ± 0.01	0.00 ± 0.01	0.01 ± 0.01

Table S6

Relative ratio of the peak areas of high-KCl-induced contraction in vehicle, FPL64176, P_4 + vehicle, and P_4 + FPL64176 groups

Time (min) Group (n = 6, mean ± SD)	-5-0	0-5	5-10	10-15	15-20	20-25	25-30	30-35	35-40
High-KCl + FPL64176	1.10 ± 0.06	1.44 ± 0.11	1.73 ± 0.13	1.90 ± 0.15	2.02 ± 0.17	2.10 ± 0.19	2.16 ± 0.19	2.18 ± 0.18	2.19 ± 0.17
High-KCl + Vehicle	1.10 ± 0.07	1.20 ± 0.07	1.19 ± 0.12	1.18 ± 0.15	1.17 ± 0.18	1.16 ± 0.19	1.15 ± 0.20	1.15 ± 0.21	1.14 ± 0.21
High-KCl + P_4 + FPL64176	1.09 ± 0.08	0.95 ± 0.09	0.74 ± 0.10	0.61 ± 0.11	0.54 ± 0.10	0.63 ± 0.10	0.90 ± 0.07	1.09 ± 0.04	1.21 ± 0.04
High-KCl + P_4 + Vehicle	1.02 ± 0.03	0.94 ± 0.11	0.73 ± 0.14	0.62 ± 0.15	0.55 ± 0.15	0.48 ± 0.15	0.45 ± 0.15	0.42 ± 0.15	0.40 ± 0.14
